# Housekeeping and tissue-specific genes in mouse tissues

**DOI:** 10.1186/1471-2164-8-127

**Published:** 2007-05-22

**Authors:** Kouame E Kouadjo, Yuichiro Nishida, Jean F Cadrin-Girard, Mayumi Yoshioka, Jonny St-Amand

**Affiliations:** 1 Functional Genomics Laboratory, Molecular Endocrinology and Oncology Research Center, Laval University Medical Center (CHUL), Department of Anatomy and Physiology, Laval University, 2705 Boulevard Laurier, Québec, G1V 4G2, Canada

## Abstract

**Background:**

This study aims to characterize the housekeeping and tissue-specific genes in 15 mouse tissues by using the serial analysis of gene expression (SAGE) strategy which indicates the relative level of expression for each transcript matched to the tag.

**Results:**

Here, we identified constantly expressed housekeeping genes, such as eukaryotic translation elongation factor 2, which is expressed in all tissues without significant difference in expression levels. Moreover, most of these genes were not regulated by experimental conditions such as steroid hormones, adrenalectomy and gonadectomy. In addition, we report previously postulated housekeeping genes such as peptidyl-prolyl cis-trans isomerase A, glyceraldehyde-3-phosphate dehydrogenase and beta-actin, which are expressed in all the tissues, but with significant difference in their expression levels. We have also identified genes uniquely detected in each of the 15 tissues and other tissues from public databases.

**Conclusion:**

These identified housekeeping genes could represent appropriate controls for RT-PCR and northern blot when comparing the expression levels of genes in several tissues. The results reveal several tissue-specific genes highly expressed in testis and pituitary gland. Furthermore, the main function of tissue-specific genes expressed in liver, lung and bone is the cell defence, whereas several keratins involved in cell structure function are exclusively detected in skin and vagina. The results from this study can be used for example to target a tissue for agent delivering by using the promoter of tissue-specific genes. Moreover, this study could be used as basis for further researches on physiology and pathology of these tissues.

## Background

Housekeeping genes are constitutively expressed in all tissues to maintain cellular functions [1]. Moreover, they are presumed to produce the minimally essential transcripts necessary for normal cellular physiology [2]. In the validation of GeneChips using real-time RT-PCR, a standard curve of a reference housekeeping gene is thus sufficient for the quantification of multiple genes in a tissue. This is especially advantageous for the validation of GeneChip results because multiple genes are quantified in one tissue sample [3]. However, the expression level of the housekeeping genes may vary among tissues or cells and may change under certain circumstances. On the other hand, the highly specific tissue expression of a gene indicates that the gene performs a tissue-specific function. A feature of housekeeping genes is that, in comparison to tissue-specific genes, they evolve on average more slowly [4]. In addition, the methylation is one of the major epigenetic modifications of DNA in mammalian cells, regulating tissue-specific genes as well a housekeeping genes [5]. Furthermore, the differences in the chromatin features between specific genes and housekeeping genes indicate the involvement of chromatin organisation in the control of gene expression [6]. In addition, despite the most fundamental characteristics of housekeeping genes and specific genes, no previous study has quantified the expression level of the housekeeping genes and the tissue-specific genes in a variety of tissues. Using serial analysis of gene expression (SAGE) strategy which indicates the relative level of expression of each transcript matched to a tag, this study represents, to our knowledge, the first characterization of the tissue-specific genes and housekeeping genes in an extensive array of tissues and organs. Using SAGE method, previous studies have characterized the transcriptome of numerous tissues such as uterus [7], adipose tissue [8,9], skeletal muscle [10], hypothalamus, cerebral cortex and pituitary gland [11,12].

The invention and application of SAGE method have paralleled those of microarray/chip technologies. Whereas hybridization-based technologies may allow for shorter detection times and high throughput expression analysis, the SAGE method not only identifies unknown genes but also quantifies the gene expression level relatively to the total mRNA population. Indeed, the SAGE method can be performed to accurately measure the abundance of both known and novel transcripts on global scale [13]. This method is ideal to analyze a large number of transcripts in a given tissue, and it allows the quantitative cataloguing and comparison of expressed genes under various physiological and pathological states [13-15].

## Results

### Number of tags and tag species analyzed

A total count of 1,834,621 SAGE tags were analyzed for the 15 tissues representing 320,624 tag species. For each tissue, approximately 130,000 tags were sequenced, except for testis, ovary, mammary gland, vagina and bone which had approximately 50,000 tags.

This study has identified 1,111 ubiquitously expressed transcripts. These genes are expressed in all the tissues and therefore are likely candidates as the genes responsible for cellular maintenance also known as housekeeping genes. Among the ubiquitous genes identified, 280 genes are constantly expressed in all tissues. The rest (831 transcripts) are not expressed at the same level in all the 15 tissues. The 280 transcripts are detected at similar level in each of the 15 tissues and can be useful as a set of controls. In the Table 1, we present the expressions levels of the top 10 constantly expressed housekeeping genes, which are expressed in all tissues without significant difference in expression levels and the previously postulated housekeeping genes. To see if the ratio of the housekeeping genes changes in another condition, we have performed biologically relevant experiments. The data presented in the Table 1 have shown that most of these housekeeping genes are not only constant between intact tissues, but also between tissues that have been subjected to hormones such as dihydrotestosterone (DHT) and glucocorticoid (Gcc), as well as gonadectomy (GDX) and adrenalectomy (ADX) conditions. We have investigated the effects of GDX and DHT in prostate, mammary gland, uterus, vagina, adipose tissue and skeletal muscle as well as the regulation of ADX and Gcc in lung, hypothalamus and pituitary gland. Only ribosomal protein L37 and leukocyte receptor cluster (LRC) member 8 were regulated by GDX compared to the intact mice in mammary gland and prostate, respectively. On the other hand, only the expression level of glyceraldehyde-3-phosphate dehydrogenase (GAPDH), a previously postulated housekeeping gene, was regulated by DHT in adipose tissue and uterus compared to GDX mice. Remarkably, eukaryotic translation elongation factor 2 (eEF-2) was highly and constantly expressed in all the investigated tissues. Despites their moderate levels of expression, proteasome (prosome, macropain) 26 subunit non-ATPase 4, eukaryotic initiation factor 3 (eIF3), and ribosomal protein L38 are constantly expressed in all the tissues. We have also identified three RIKEN cDNAs constantly expressed in all the tissues. The results have also shown that, the previously postulated housekeeping genes namely peptidyl-prolyl cis-trans isomerase A (PPIase), GAPDH, beta-actin are not constantly expressed in all the tissues.

### Most expressed tissue-specific genes in the 15 tissues

We have also identified the genes uniquely detected in each of the 15 tissues. These genes are critical for the specific functions that characterize and distinguish the testis, prostate, ovary, mammary gland, uterus, vagina, skin, liver, adipose tissue, lung, bone, skeletal muscle, cerebral cortex, hypothalamus, and pituitary gland. The specific genes are expressed in only one tissue. Table 2 presents the transcripts exclusively detected in the male sexual organs. Obviously, the levels of gene expression in these tissues were statistically different from the other tissues. The current study shows 61 tissue-specific transcripts in the testis. Despite their moderate expression in the testis, some transcripts such as dipeptidase 3, ankyrin repeat domain 5, and ubiquitin-conjugating enzyme E2N are exclusively found in the testis. In contrast, only 7 transcripts are detected in the prostate as specific genes. Noteworthy, seminal vesicle protein secretion 2 is both highly and exclusively detected in the prostate. According to the transcripts specifically detected in the female sexual organs (Table 3), most of the tissue-specific genes are expressed at lower quantity in the ovary, mammary gland, uterus and vagina in comparison to the majority of specific genes expressed in the male sexual organs. Remarkably, proline-rich acidic protein 1 and the novel transcript with the sequence tag CTGTATTTGGG are both highly and uniquely detected in the uterus and the vagina, respectively. The Table 4 shows the transcripts exclusive to the skin, liver, adipose tissue, lung, bone and skeletal muscle. The majority of tissue-specific genes expressed in the skin are involved in cell structure. Keratin associated protein 8-1 is more expressed than the other tissue-specific genes detected in the skin. We report 26 transcripts exclusively detected in the liver. Moreover, two tissue-specific genes namely albumin 1 and alpha microglobulin/bikunin are also highly expressed in the liver. Despite their low expression in the liver, major urinary protein 1, solute carrier family 27 (fatty acid transporter) member 2 and silica-induced gene 111 are also specifically detected. Furthermore, the tissue-specific transcripts expressed in the lung and the bone are involved in cell defence and we have observed the high expression of the tissue-specific genes such as proteoglycan 2 bone marrow and solute carrier family 4 (anion exchanger) member 1 in the bone. The adipose tissue and the skeletal muscle expressed less tissue-specific genes than the other tissues. Only two transcripts namely leptin and lectin galactose binding soluble 12 are reported as candidate specific genes in the adipose tissue. The transcripts such as myosin light chain phosphorylatable fast skeletal muscle and tropomyosin 3 gamma are both moderately and uniquely detected in the skeletal muscle. Among the brain areas investigated, the pituitary gland expressed more tissue-specific transcripts (Table 5). In addition, the majority of these genes are also highly expressed in this gland. We found 3 genes expressed in cerebral cortex, which were not detected in any of the other 14 tissues. In the hypothalamus, RIKEN cDNA A230109K23 gene is both exclusively and highly detected. To see if the tissue-specific genes were detected in other tissues, we have compared our results to the public data deposited in Gene Expression Omnibus (GEO, NCIBI). The public data have confirmed the exclusive detection of tissue-specific genes in each tissue. The transcripts which were specifically detected in the 15 intact tissues were not expressed in other mouse tissues such as embryonic fibroblasts, heart, thymus, bladder, kidney, spleen and pancreas (Table 2 to 5). In addition, using UniGene and EST expression information in the public databases, the normalized expression (%) of intestine is shown and is compared to our results in Table 2 to 5. All the genes mentioned for the uterus, as well as carbonic anhydrase 1, retinol binding protein 2 cellular and keratin complex 1 acidic gene 13 were also expressed in the intestine tissue.

## Discussion

### The most abundant housekeeping genes

Quantitative gene expression data are often normalized to the expression levels of control or so-called housekeeping genes. An inherent assumption in the use of housekeeping genes is that expression of the genes remains constant in the tissues under investigation. Housekeeping genes do not vary in their expression levels during cell development, treatment, or disease state anomalies [16]. About 40 years ago, housekeeping genes were simply defined as those genes that are always expressed [1], also known as ubiquitous genes. However, for many experimental applications, we must make the difference between the housekeeping genes which are constantly expressed in all the tissues and the ubiquitous genes. According to the current study, the ubiquitous genes also previously called housekeeping genes such as PPIase, GAPDH, and beta-actin are expressed in all tissues with significant difference in their expression levels. On the other hand, the housekeeping genes eEF-2, ribosomal protein L37 and L38, proteasome (prosome, macropain) 26 subunit non-ATPase 4, eIF3, hypothetical protein, LRC member 8 also known as interleukin-8 receptor (IL-8R), and the RIKEN cDNA C030036P15, 1500016L11 and 2410104I19 genes are constantly expressed in all the tissues investigated. Our results are consistent with the previous report that ribosomal protein L37, proteasome and hypothetical protein are expressed at the same level in 11 human adult and fetal tissues [17]. In addition, except LRC member 8 and the RIKEN cDNAs, the transcripts reported as housekeeping genes in this study have also been previously presented as maintenance genes in a study investigating 11 human adult and fetal tissues [17]. Using the SAGE method, Velculescu et al also identified the same ubiquitous transcripts among nearly 1000 transcripts expressed at more than or equal to 5 copies par cell from 19 human tissues [18]. However, they did not apply the statistics to identify the constantly expressed genes. In the current study, we report eEF-2 as the housekeeping gene which is abundantly and constantly transcribed in all of the 15 tissues. Indeed, eEF-2 is known to be required for elongation in most eukaryotes [19]. Since eEF-2 does not vary in relative abundance in different tissues, this gene can be used as a standard or internal control. The constitutive expressions of ribosomal proteins L37 and L38 were observed in all the tissues. Our results are also consistent with the report that ribosomal protein L37 is constitutively expressed during transitions from quiescence to active cell proliferation or terminal differentiation in all tissues in rat and human [20]. Another study has shown that ribosomal protein L37 ranks amongst the fifteen most highly expressed housekeeping genes [21]. Furthermore, the present study identified LRC member 8 and eIF3 as housekeeping genes. LRC is known to play an important role in cell defence in all tissues. In mammals, the cell surface receptor encoded by LRC member 8 regulates the activity of lymphocytes and natural killer cells in order to provide protection against pathogens and parasites [22]. Previous studies have presented interleukin-12 receptor beta 2 [18] and interleukin-1 receptor-associated kinase [17] as ubiquitously expressed genes. No previous study had investigated LCR member 8 as a housekeeping gene or internal control. According to this study, we suggest for the first time interleukin-8 receptor as housekeeping gene or internal control since this transcript is ubiquitously expressed in all the tissues investigated. Proteasome (prosome, macropain) 26 subunit non-ATPase 4, the major proteolytic machinery responsible for degradation of both normal and damaged proteins has been identified as housekeeping gene. Our results are in agreement with the knowledge that proteasome is a multicatalytic complex found in all eukaryotic cells [23], and also with the report that proteasome ranks amongst the fifteen most constant housekeeping genes [21]. Therefore, our results are consistent with previous reports showing that housekeeping genes are generally involved in a variety of basic cellular functions, including intermediary metabolism, transcription, translation, cell signalling/communication and cell structure/motility [21]. In addition, the current study has identified three EST RIKEN cDNAs (C030036P15, 2410104I19 and 1500016L11) as housekeeping genes. We have also investigated three previously postulated housekeeping genes namely PPIase, beta-actin, and GAPDH [24] which are expressed with significant difference according to the current study. Our study is in agreement with the previous report that PPIase, beta-actin and GAPDH are maintenance genes expressed in 11 human adult and fetal tissues but are not expressed at the same level in these tissues [17]. GAPDH was especially highly expressed in the skeletal muscle. Beta-actin was also differentially expressed between the tissues. According to the present study, PPIase is more constantly expressed in all the tissues than beta-actin and GAPDH. This result can explain the previous report that PPIase is a better internal control than beta-actin and GAPDH [25].

### Specific genes expressed in the male sexual organs

According to this study, the tissue-specific genes were observed in higher proportion in testis than any other tissues. The top 8 most abundant tissue-specific transcripts, namely protamine 2, transition protein 1, t-complex-associated testis expressed 3, the novel transcript GTGCCAGGAGA, tubulin alpha 3, diazepam binding inhibitor-like 5, lactate dehydrogenase 3 C chain sperm specific, and outer dense fiber of spermtails 2 are also the top 8 most abundant transcripts in this organ. These tissue-specific genes are all expressed in the specific stage of spermatogenesis. Other tissue-specific genes such as spermatogenic Zip 1 [26] and glyceraldehydes 3-phosphate dehydrogenase-spermatogenic [27], are expressed only during spermatogenesis. Translin associated factor X (Tsnax) interacting protein 1 is highly expressed in testis and in germ cells, suggesting a possible role in spermatogenesis [28]. Meanwhile, four-and-a-half-Lim-domain 5 is a LIM-only protein expressed exclusively in round spermatids [23], while kinesin family member 2b [29] and dipeptidase 3 [30] are expressed only in testis. The current study also reports the expression of testis specific genes such as ATPase class I type 8b member 3 which has hydrolase and phospholipid-translocating ATPase activity, and ATP-dependent aminophospholipid transporter which is exclusively expressed in the acrosomal region of spermatozoa [31]. In mammals, thioredoxins (trx) are generally ubiquitously expressed in all tissues, with the exception of sperm-specific trx (sptrx) which is exclusively detected in sperm cells [32]. The specific functions of these transcripts involved in spermatogenesis, explain the exclusive expression of these transcripts in the testis.

We have identified some prostate specific genes such as microseminoprotein (beta-MSP), seminal vesicle protein secretion 2, seminal vesicle antigen (SVA) and mucin 10 (MUC10) which are involved in protein secretion, cell signalling and spermatogenesis. In addition, one novel transcript was observed to be solely expressed in the prostate. Beta-MSP, also known as prostate secretory protein of 94 amino acids (PSP94), is an abundant secretory protein of the prostate gland and is generally considered to be prostate tissue-specific [33]. Results from western and northern-blot analyses for various tissues had previously indicated that the seminal vesicle is the sole organ producing SVA which is able to induce autoantibody formation [34]. MUC10, the submandibular salivary gland (SMG) mucin which is the primary histodifferentiation product of submandibular epithelia, was observed as tissue-specific gene to the prostate in our study. MUC10 is a type 1 integral membrane protein with a disintegrin and metalloprotease domain (ADAM29) precursor involved in spermatogenesis and fertilization [35]. Other prostate tissue specific identified are CUB and zona pellucida-like domains 1 (Cuzd1), and serine peptidase inhibitor Kazal type 3. In mouse, the expression of a secretory protease inhibitor is constitutive in the pancreas but stimulated by testosterone in ventral prostate, coagulated gland and seminal vesicle [36]. Cuzd1 was identified as highly and predominantly expressed gene in mouse epididymis using a cDNA microarray [37]. Since prostate-specific antigen (PSA), also known as gamma-seminoprotein, is a serine protease produced and secreted abundantly by prostate cancer cells [38], we did not detect PSA in the current results on normal prostate. Probasin, previously postulated as tissue-specific gene in the prostate [39], was abundantly expressed in the prostate (1925 tags) and was also found in the liver (282 tags) according to the present study. These results may have important repercussion, since prostate tissue-specific genes expression is crucial for driving potentially therapeutic genes to target specifically to the prostate [39].

### Specific genes expressed in the female sexual organs

Only two transcripts with unknown function and a novel transcript with sequence tag GTCAACACAGG were specifically detected in ovary. We report two transcripts, namely glycosylation cell adhesion molecule 1 (Glycam1) and the novel transcript with sequence tag TCCGGAGAAAA, which are expressed only in the mammary gland. Glycam1 has a protein binding activity and previous studies have reported that prolactin induced Glycam1 expression in primary mammary epithelial cell of mice [40].

In uterus, three transcripts namely proline-rich acidic protein 1 (Prap1), hydroxysteroid 11-beta dehydrogenase 2 (Hsd11b2) and chloride channel calcium activated 3 (clca3) were characterized as tissue-specific. Northern analyses have demonstrated that Prap1 also known as pregnancy-specific uterine protein expression is limited to the pregnant uterus [41]. In the rat, the enzyme Hsd11b2 converts the glucocorticoid corticosterone into receptor-inactive 11 dehydrocorticosterone, thereby allowing preferential access of aldosterone to mineralocorticoid receptors. The effects of glucocorticoids are thus critically regulated by the intracellular enzyme Hsd11b2 which was shown here to be highly expressed in the uterus [42]. In addition, the information from UniGene and EST expression has shown that the transcripts identified in uterus are also expressed in intestine tissue.

Two novel transcripts were observed to be tissue-specific to the vagina. Furthermore, the keratins such as keratin intermediate filament 16a, keratin complex 1 acidic gene 13 and keratin complex 2 basic gene 6 g, members of a family of fibrous structural proteins were also detected in high proportions in the vagina. Surprisingly, retinol binding protein 2, which functions in the intracellular transport of retinol, is detected only in vagina. According to the UniGene and EST databases, retinol binding protein 2 is also expressed in intestine tissue.

### Specific genes expressed in the skin, liver, adipose tissue, lung, bone and skeletal muscle

The majority of transcripts specifically detected in the skin represent keratin. Moreover, the S100 calcium binding protein 17 and G protein-coupled receptor family C group 5 member D, both involved in cell signalling, were also specific to the skin. In addition, lymphocyte antigen 6 complex locus G6C, involved in cell defence, was also exclusively observed in the skin.

The transcripts exclusive to the liver are involved in several functions such as cell defence, protein and steroid hormone synthesis, as well as transport and amino acid metabolism. We report in the current study that inter-alpha-trypsin inhibitor-4 (Itih-4) was exclusively detected in the liver. This result is in agreement with the report that Itih-4 is a liver-restricted member of the serine protease inhibitors family, with diverse functions such as anti-apoptotic action and matrix stabilization molecule that are important throughout development [43]. The present results also suggest that betaine-homocysteine methyltransferase (BHMT), cytochrome P450 family 2 subfamily d polypeptide 26 (Cyp2d26), microglobulin/bikunin precursor (AMBP) and plasminogen are solely expressed in the liver amongst the included organs. Previous studies have shown that BHMT [44] and Cyp2d26 [45] were expressed only liver and kidney, whereas strong expression of AMBP has been observed in developing hepatocyte, pancreas, kidney and gut [46]. The expression of plasminogen mRNA from hepatocyte is dependent on the cell density and stimulation [47] and is solely expressed in liver. Two mouse plasma proteins involved in cell defence, namely murinoglobulin 2 and kininogen 2 were exclusively expressed in the liver. Murinoglobulin is characterized as a single-chain proteinase inhibitor [48], while kininogen is known as a major acute phase protein whose levels increase 10–20 fold in response to an inflammatory challenge [49]. Traditionally, 4-hydroxyphenylpyruvic acid dioxygenase (HPD) has been considered as an enzyme primarily expressed in liver and to a lesser extent in kidney [50]. This previous study is agreement with the current study in which HPD is exclusively detected in the liver.

Two transcripts, leptin and lectin galactose binding soluble 12 (Lgals12), were exclusively detected in adipose tissue. Leptin is a peptide hormone produced predominantly by white adipose tissue. Beside its key role in the regulation of food intake and energy expenditure, leptin is also involved in the pathogenesis of inflammatory and autoimmune disease [51]. On the other hand, Lgals12 also known as galectin-12 is preferentially expressed in mouse preadipocytes and is up-regulated when preadipocytes undergo cell cycle arrest [52].

The transcripts specific to the lung are involved in cell defence, except claudin 18 which participates in cell signalling. The surfactant-associated proteins A (SP-A) and SP-D are members of a family of collagenous host defence lectins, designated collectins. The lung is the main site of SP-A and SP-D synthesis [53]. They are considered to be molecules of the innate immune system involved in the first line of defence of mucosal surfaces, especially in lung [53]. Another specific transcript is the palate lung and nasal epithelium clone (Plunc), also renamed Splunc1, which is a small secreted protein expressed in the oropharynx and upper airways of humans, mice, rats and cows. The members of the PLUNC family may be involved in the innate immune responses in regions of the mouth, nose and lung, which are sites of significant bacterial exposure [54]. Another transcript exclusively detected in the lung is secretoglobin family 3A member 1 which is thought to play a role in inflammation and/or epithelial cell differentiation in the lung. The mRNA encoding for this gene is expressed predominantly with low level in terminals bronchioles [55].

The transcripts exclusively detected in the bone are involved in transport, cell structure, signalling and defence. The tissue-specific genes ascribed to proteoglycan 2 bone marrow and solute carrier family 4 (anion exchanger) member1 are highly and solely expressed in the bone. The other exclusive transcripts such as proteinase 3, neutrophil elastase, eosinophil peroxidase, cathepsin G and carbonic anhydrase 1 are involved in cell defence. Evidence from northern analysis has shown that proteinase 3 expression is primarily confined to the promyelocytic/myelocytic stage of bone marrow development [56]. The highly related serine protease known also as neutrophil elastase, proteinase 3 and cathepsin G are exclusively detected in promyelocytes and packaged in azurophil granules [57,58], whereas carbonic anhydrase 1 is expressed in adult human and mouse erythroid cells and colon epithelia, from two distinct promoters [59]. Another transcript exclusively expressed in bone matches to haematopoietic cell-specific transmembrane-4 (HTm4), member of a family of membrane-spanning 4-domain proteins. It is known that HTm4 is expressed in hematopoietic tissue and is tightly regulated during the differentiation of hematopoietic stem cells [60].

In the current study, the transcripts exclusively detected in skeletal muscle are novel transcripts except tropomyosin 3 gamma (Tpm3) and myosin light chain phosphorylatable fast skeletal muscle (MLC2) which are involved in cell structure. In the mice, the Tpm3 mRNA is found exclusively in the skeletal muscle but not in the cardiac tissue at any development stage, whereas, in human, Tpm3 is found in both adult heart and skeletal muscle [61,62]. The specificity of MLC2, reported in the current study is in agreement with the previous study showing this gene expressed specifically in skeletal muscles of new-born and adult mice as well as rats [63].

### Specific genes expressed in the brain

The current study reports that solute carrier family 17 (sodium-dependent inorganic phosphate cotransporter) member 7, also known as vesicular glutamate transporters (Vglut1), and synaptic vesicle glycoprotein 2 b (SV2B) as well as gene model 748 were exclusively expressed in cerebral cortex. This result is consistent with the report that Vglut1 mRNA is strongly expressed in the hippocampus and cerebellar cortex [64]. Vglut1 has been observed to play an unanticipated role in membrane trafficking at the nerve terminal [64]. SV2B is a protein highly related to a family of transporters. SV2B expression was observed to change during development; it is more widely expressed in the immature brain and is found in cells that have yet to establish synaptic contacts. SV2B is expressed in cerebral cortex, therefore further studies on the expression pattern of SV2B are needed to investigate the consistency with its function as a specific neurotransmitter transporter [65].

We report that cocaine and amphetamine regulated transcript (CART), calbindin 2, hypocretin and RIKEN cDNA A230109K23 gene were exclusively detected in the hypothalamus. The protein calbindin 2 buffers intracellular calcium is speculated to be involved in the integration of neuronal signalling. CART encodes a hypothalamic neuropeptide precursor protein which has been identified and characterized in rat brain and later in human brain [66,67]. Furthermore, hypocretin-1 and -2 (Hcrt-1 and Hcrt-2), also referred to as orexin-A and -B, are neuropeptides synthesized by a few thousand neurons in the lateral hypothalamus. Studies have hinted at a role of hypocretins in driving drug-seeking through activation of stress pathways in the brain [68]. Further investigation should be made in the characterization of RIKEN cDNA A230109K23.

The transcripts exclusively expressed in the pituitary gland were also highly expressed in this gland. Except growth hormone, pro-opiomelanocortin-alpha, prolactin, luteinizing hormone beta (LH beta) and thyroid stimulating hormone beta subunit (TSH beta) also known as thyrotropin beta which are involved in cell signaling, the other specific genes to this gland had no match in public databases and therefore may represent novel transcripts. LH beta is essential for ovulation and reproductive fitness, and is well known to be synthesized specifically in pituitary gonadotropes [69]. In addition, this glycoprotein is essential for ovarian follicular development, maturation of the oocyte, steroidogenesis and ovulation. In males, LH beta is involved in regulation of steroidogenesis in the Leydig cells [70]. TSH is an anterior pituitary glycoprotein hormone which modulates thyroid hormone production by the thyroid gland. TSH is constituted of two subunits: the alpha subunit is held in common with the gonadotropins luteinizing hormone and follicle-stimulating hormone, and the beta subunit which is unique and confers biological specificity to the intact hormone [71].

## Conclusion

Using SAGE strategy, this study has shown the housekeeping genes and the tissue-specific genes expressed in 15 intact tissues. The identified housekeeping genes can represent appropriate controls for RT-PCR and northern blot when comparing the expression levels of genes in several tissues. Several transcripts exclusively detected in a tissue are known to be tissue-specific genes according to previous studies. Furthermore, we have identified several new tissue-specific genes. These genes show well the specialty and particularity of each tissue. The tissue-specific genes can be used as a targeting agent in order to reach a particular tissue/organ. In addition, the current data can contribute significantly to comparative genomics in general and gene expression and regulation among different mouse tissues. Further studies will be needed to investigate other tissues to confirm the specific or housekeeping gene expressions. In addition, this study can serve as a basis for future studies on the novel transcripts and the transcripts with unclear functions despite their tissue specificity.

## Methods

C57BL6 mice (12–15 week old) were obtained by Charles River Laboratories (St. Constant, Québec). They were housed in an air-condition room (19–25°C) with controlled lighting from 07:15 to 19:15 h and were given free access to food (Lab Rodent Diet No. 5002) and water. The GDX and ADX groups had surgery 7 days before death. The intact, GDX and ADX groups received vehicle solution (0.4% (w/v) Methocel A15LV Premium/5% ethanol) 24 hours before sacrifice. DHT (0.1 mg) was injected 3 h prior to killing in GDX+DHT groups. ADX mice received sodium chloride (0.9 g/dl) in their drinking water after the surgery. Gcc (corticosterone, 0.1 mg per mouse) was subcutaneously injected into ADX mice, and the tissues were harvested 3 h after the injection. All animal experimentation was conducted in accord with the requirements of the Canadian Council on Animal Care. All the tissues were from male mice except for female sexual tissues. The tissues were dissected from 15–51 mice, frozen in liquid nitrogen and stocked at -80°C until analysis.

Total RNA was isolated from tissues by using the RNA extraction kit (TRIzol Reagent, Invitrogen Canada Inc., Burlington, ON). Approximately 5 μg of mRNA was extracted with Oligotex mRNA Mini Kit (Qiagen Inc., Mississauga, ON). The SAGE method was performed as previously described [13,14]. In brief, double-strand cDNA was synthesized from the mRNA using a biotinylated (T)_18 _primer and cDNA synthesis kit (Invitrogen Canada Inc.). The cDNA libraries were digested with the restriction enzyme NlaIII (New England Biolabs Inc., Pickering, ON). The 3'-terminal cDNA fragments were captured using streptavidin-coated magnetic beads (Dynal, Biotech LLC, Brown Deer, WI). After ligation of 2 annealed linker pairs, the cDNA fragments were digested with BsmFI (New England Biolabs Inc.). The blunting kit from Takara Bio Inc. (Otsu, Japan) was used for the blunting and ligation of the two tag populations. The resulting ligation products were amplified by PCR and digested with NlaIII. The band containing the ditags was extracted from the 12% polyacrylamid gel. Using T4 ligase (Invitrogen Canada Inc.), the ditags were self-ligated to form concatemers that were cloned into SphI site of pUC19. White colonies were screened by PCR and agarose gel to select long inserts for automated sequencing (Applied Biosystems 3730, Foster City, CA). The data discussed in this publication have been deposited in NCBIs Gene Expression Omnibus [72] and are accessible through GEO Series accession number GSE5915. The sequence and occurrence of each of the transcript tags has been determined using the software SAGEana.pl, an updated version of SAGEparser.pl [73]. To identify the transcripts, we have generated a SAGEmap of 11 bp tags by the script SAGEmap.pl using the NCBI 10 bp tags SAGEmap, as well as the UniGene Clusters and mitochondrial mRNA sequences. The tag sequences must perfectly match at the last Nla*III *restriction site (CATG) at the 3' end of a given transcript. To overcome the lower quality of some EST sequences, the tags that did not identify a well-characterized mRNA were required to match at least two ESTs in the same UniGene Cluster including one EST with a known polyA tail. To identify the transcripts, the sequences of 15 bp SAGE tags were matched with public databases. The tag numbers normalized by 100000 are shown in Tables 1, 2, 3, 4, 5.

The SAGE method has very good reproducibility [73]. However, several factors can affect this reproducibility such as the failure to provide relevant matching statistics. When a tag matches multiple genes, it is impossible to know the number of copies which are contributed by each gene since the matching stastitic is giving by the mixture of all contributing genes. However, using a 15 bp (CATG + 11 bp) tag, the SAGEparser program decreases the number of multiple matches and increases the number of tags which uniquely identify a transcript [73]. Therefore, the tags matching multiple genes were excluded from the current report. We used the comparative count display (CCD) test to identify the transcripts that were significantly (p ≤ 0.05) differentially expressed between the groups with more than 2-fold change. CCD test performs a key-by-key comparison of two key-count distributions by generating a probability that the frequency of any key in the distribution differs by more than a given fold factor from the other distribution. This statistical test has already been described elsewhere [74]. We have used the web site source at Stantford [75] to add the comparison of the tissue-specific genes with the UniGene and EST expression database information.

## Authors' contributions

KEK has participated in the SAGE analyses including the bioinformatic analysis and wrote the paper with assistance from YN and JFCG. MY contributed to the conception and design of the project and drafted the manuscript. JSA directed the study, contributed to the conception and design of the project, analysis and interpretation of the data and drafted the manuscript. All the authors edited, read and approved the final manuscript.

## Figures and Tables

**Table 1:** Housekeeping genes expressed in 15 tissues

					Tissues (total tags count sequenced)													
				
Tag	**Testis **(56909)	**Prostate **(143559)	**O vary **(56154)	**Mammary gland **(40546)	**Uterus **(178824)	**Vagina **(50819)	**Skin **(161093)	**Liver **(170384)	**Adipose tissue **(158218)	**Lung **(159843)	**Bone **(74859)	**Skeletal muscle **(135530)	**Cerebral cortex **(161221)	**Hypothalamus **(165981)	**Pituitary gland **(128480)	**Regulation by GDX, DHT, ADX or Gcc**	Description [UniGene cluster, GenBank Accession no.]	General function
**Housekeeping genes constantly expressed**																		
GCCAAGTGGAG	109	87	*216*	94	141	203	147	154	184	159	123	146	96	129	111		eukaryotic translation elongation factor 2 [Mm.334671, BB705125]	Protein synthesis
GATTCCGTGAG	79	85	186	92	128	191	161	81	106	127	126	85	98	147	88	GDX^m^	ribosomal protein L37 [Mm.10474, AK012544]	Protein synthesis
CCTCGGAAAAT	9	10	21	10	21	32	19	11	22	12	25	35	20	25	28		ribosomal protein L38 [Mm.238817, BC055346]	Protein synthesis
GTAGCTCACAA	19	15	27	17	22	20	14	17	16	24	25	13	12	17	28		hypothetical protein [Mm.302962, BB767941]	Protein secretion
TAACTGGAGGA	30	10	27	10	10	28	19	10	16	28	15	7	22	28	12	GDX^p^	leukocyte receptor cluster (LR C) member 8 [Mm.22831, BC066768]	Cell defence
GTGGTGCACAC	19	10	11	10	9	24	11	16	14	18	15	13	15	29	24		RIKEN cDNA C030036P15 gene [Mm.36291, NM_172757]	Protein binding
CTCAACAGCAA	5	9	27	15	12	20	17	14	17	14	16	14	16	10	15		eukaryotic translation initiation factor 3, subunit 5 (epsilon) [Mm.182962, NM_025344]	Protein synthesis
GGTGATGAGGA	4	9	7	15	9	22	13	12	25	11	15	24	10	19	10		RIKEN cDNA 1500016L11 gene [Mm.295670, NM_026885]	Cell division
GGGGCTCTGGC	16	9	20	10	10	12	10	16	16	8	25	21	8	7	6		proteasome (prosome, macropain) 26S subunit, non-ATPase, 4 [Mm.2261, NM_008951]	Cell defence
GCCTCAGAGAC	7	5	9	10	10	14	9	5	13	14	7	11	18	19	13		RIKEN cDNA 2410104I19 gene [Mm.29965, NM_133691]	translational initiation regulation
Previously postulated housekeeping genes																		
GAGCGTTTTGG	63	**49**	130^a^	84	119^a^	177^a^	127^a^	54	296^a^	153^a^	178^a^	93	238^a^	226^a^	61		peptidyl-prolyl cis-trans isomerase A (PPIase) [Mm.342899, XM_122578]	Protein synthesis
GCCTCCAAGGA	**16**	47^a^	98^a^	114^a^	70^a^	175^a^	149^a^	159^a^	366^a^	64^a^	159^a^	2332^a^	309^a^	267^a^	97^a^	DHT^at, u^	glyceraldehyde-3-phosphate dehydrogenase [Mm.303486, XM_285250]	Sugar metabolism
GATACTTGGAA	28^a^	90^a^	130^a^	171^a^	179^a^	132^a^	107^a^	79^a^	144^a^	295^a^	296^a^	**8**	102^a^	86^a^	62^a^		actin, beta, cytoplasmic [Mm.297, BB836462]	Cell structure
CCCTGAGTCCA	52^a^	**18**	159^a^	163^a^	102^a^	168^a^	174^a^	88^a^	134^a^	268^a^	444^a^	30	100^a^	86^a^	31		actin, beta, cytoplasmic [Mm.297, NM_007393]	Cell structure

^a^Significant difference vs.no.of tags shown in bold (p < 0,05). The tag numbers were normalized by 100000.

Abbreviation: GDX, gonadectomy; DHT, dihydrotestosterone; ADX, adrenalectomy; Gcc, glucocorticoids;^m^, mammary gland; ^p^, prostate; ^at^, adipose tissue; ^u^,uterus

**Table 2:** Specific genes expressed in the male sexual organs

																Tissues from GEO and accession number									
																			
Tag	**Testis**	**Prostate**	Ovary	Mammary gland	Uterus	Vagina	Skin	Liver	Adipose tissue	Lung	Bone	Skeletal muscle	Cerebral cortex	Hypothalamus	Pituitary gland	Mouse embryonic fibroblasts GSM7759	Heart normal GSM106587	Thymus normal GSM106587	Bladder normal GSM106599	Kidney normal GSM56242	Spleen normal GSM106591	Pancreas normal GSM 106592	Intestine UniGene and EST expression (%)	Description [UniGene cluster, GenBank Accession No]	General function
**Testis**																									
TCGATGTCTGA	**1221**	0^a^	4^a^	0^a^	0^a^	0^a^	0^a^	1^a^	1^a^	0^a^	0^a^	0^a^	0^a^	0^a^	0^a^	0^a^	0^a^	0^a^	0^a^	0^a^	0^a^	0^a^	0	protamine 2 [Mm.325769, NM_008933]	Cell signaling
AGGACATCAGA	**601**	0^a^	2^a^	0^a^	0^a^	0^a^	0^a^	0^a^	0^a^	0^a^	0^a^	0^a^	0^a^	0^a^	0^a^	0^a^	0^a^	0^a^	0^a^	0^a^	0^a^	0^a^	0	transition protein 1 [Mm.661, NM_009407]	Cell signaling
AAACAGAGTCT	**391**	0^a^	2^a^	0^a^	0^a^	0^a^	0^a^	0^a^	1^a^	0^a^	1^a^	0^a^	0^a^	0^a^	0^a^	0^a^	0^a^	0^a^	0^a^	0^a^	0^a^	0^a^	0	t-complex-associated testis expressed 3 [Mm.272173, NM_011560]	Cell signaling
GTGCCAGGAGA	**241**	1^a^	0^a^	0^a^	0^a^	0^a^	0^a^	0^a^	0^a^	0^a^	0^a^	0^a^	0^a^	0^a^	0^a^	0^a^	0^a^	0^a^	0^a^	0^a^	0^a^	0^a^		NM	Novel transcript
GGTCTGGCTGG	**234**	0^a^	0^a^	0^a^	0^a^	0^a^	0^a^	0^a^	0^a^	0^a^	0^a^	0^a^	0^a^	0^a^	0^a^	0^a^	0^a^	0^a^	0^a^	0^a^	0^a^	0^a^	0	tubulin, alpha 3 [Mm.287784, NM_009446]	Cell signaling
GCTCCACTGGT	**227**	0^a^	0^a^	0^a^	0^a^	8	0^a^	0^a^	0^a^	0^a^	0^a^	0^a^	0^a^	0^a^	0^a^	0^a^	0^a^	0^a^	0^a^	0^a^	0^a^	0^a^	0	diazepam binding inhibitor-like 5 [Mm.347413, BC048474]	Cell signaling
ACCGCTGAGGA	**222**	0^a^	0^a^	0^a^	0^a^	0^a^	0^a^	0^a^	0^a^	0^a^	0^a^	0^a^	0^a^	0^a^	0^a^	0^a^	0^a^	0^a^	0^a^	0^a^	0^a^	0^a^	0	lactate dehydrogenase 3, C chain, sperm specific [Mm.16563, NM_013580]	Energy metabolism
TGCAACTGGCC	**201**	0^a^	0^a^	0^a^	1^a^	2^a^	1^a^	0^a^	0^a^	1^a^	1^a^	0^a^	0^a^	2^a^	0^a^	0^a^	0^a^	0^a^	1^a^	0^a^	0^a^	0^a^	0	outer dense fiber of sperm tails 2 [Mm.330116, NM_013615]	Cell defence
TTCCATCTCTG	**190**	0^a^	0^a^	0^a^	0^a^	0^a^	0^a^	0^a^	0^a^	1^a^	0^a^	0^a^	0^a^	0^a^	1^a^	0^a^	0^a^	0^a^	0^a^	0^a^	0^a^	0^a^	0	ornithine decarboxylase antizym e 3 [Mm.331200, NM_016901]	Amoni acid metabolism
TTTAGCCGAGA	**180**	0^a^	0^a^	0^a^	0^a^	0^a^	0^a^	0^a^	0^a^	0^a^	0^a^	0^a^	0^a^	0^a^	0^a^	0^a^	0^a^	0^a^	0^a^	0^a^	0^a^	0^a^	0	glyceraldehyde-3-phosphate dehydrogenase, sperm atogenic [Mm.1729, NM_008085]	Energy metabolism
TACACGAGGAT	**178**	0^a^	0^a^	0^a^	0^a^	0^a^	0^a^	0^a^	0^a^	0^a^	0^a^	0^a^	0^a^	0^a^	0^a^	0^a^	0^a^	0^a^	0^a^	0^a^	0^a^	0^a^	0	transition protein 2 [Mm.206798, NM_013694]	Cell signaling
GCCAGATACCG	**139**	0^a^	0^a^	0^a^	0^a^	0^a^	0^a^	0^a^	0^a^	0^a^	0^a^	0^a^	0^a^	0^a^	0^a^	0^a^	0^a^	0^a^	0^a^	0^a^	0^a^	0^a^	0	protamine 1 [Mm.42733, NM_013637]	Cell signaling
GATTAAAGCTT	**134**	0^a^	0^a^	0^a^	0^a^	0^a^	0^a^	0^a^	0^a^	0^a^	0^a^	0^a^	0^a^	0^a^	0^a^	0^a^	0^a^	0^a^	0^a^	0^a^	0^a^	0^a^	0	transition protein 2 [Mm.206798, NM_013694]	Cell signaling
GCGTGCTCAGA	**132**	0^a^	0^a^	0^a^	0^a^	0^a^	0^a^	0^a^	0^a^	0^a^	0^a^	0^a^	0^a^	0^a^	0^a^	0^a^	0^a^	0^a^	0^a^	0^a^	0^a^	0^a^	0	RIKEN cDNA 4921511K06 gene [Mm.251303, AK160245]	Cytokinesis, cell shape, secretion and capping
TAGCCCCTGCA	**125**	0^a^	0^a^	0^a^	0^a^	0^a^	0^a^	0^a^	0^a^	0^a^	0^a^	0^a^	0^a^	0^a^	0^a^	0^a^	0^a^	0^a^	0^a^	0^a^	0^a^	0^a^	0	outer dense fiber of sperm tails 1 [Mm.252830, NM_008757]	Cell defence
CCCTTTTTCAA	**123**	0^a^	0^a^	0^a^	0^a^	0^a^	0^a^	0^a^	0^a^	0^a^	0^a^	0^a^	0^a^	0^a^	0^a^	0^a^	0^a^	0^a^	0^a^	0^a^	0^a^	0^a^	0	sperm mitochondria-associated cysteine-rich protein [Mm.331192, NM_008574]	Sperm motility
CAGGAACACGG	**111**	0^a^	0^a^	0^a^	0^a^	0^a^	0^a^	0^a^	0^a^	0^a^	0^a^	0^a^	0^a^	0^a^	0^a^	0^a^	0^a^	0^a^	0^a^	0^a^	0^a^	0^a^		NM	Novel transcript
GTCGACCGATG	**100**	0^a^	0^a^	0^a^	0^a^	0^a^	0^a^	0^a^	0^a^	1^a^	0^a^	0^a^	0^a^	0^a^	0^a^	0^a^	0^a^	0^a^	0^a^	0^a^	0^a^	0^a^	0	meiosis expressed gene 1 [Mm.2688, CN833209]	Potein binding
CAGCTCAAGTG	**99**	0^a^	0^a^	0^a^	0^a^	0^a^	0^a^	0^a^	0^a^	0^a^	0^a^	0^a^	0^a^	1^a^	0^a^	0^a^	0^a^	0^a^	0^a^	0^a^	0^a^	0^a^	0	ATPase, C lass I, type 8B, member 3 [Mm.52511, NM_026094]	Energy metabolism, transport
CCAATTGCTAC	**95**	0^a^	0^a^	0^a^	0^a^	0^a^	0^a^	0^a^	0^a^	0^a^	0^a^	0^a^	0^a^	0^a^	0^a^	0^a^	0^a^	0^a^	0^a^	0^a^	0^a^	0^a^	0	histone H1-like protein in spermatids 1 [Mm.30482, NM_018792]	Cell division
TCGGTGCCTCT	**88**	0^a^	2^a^	0^a^	0^a^	0^a^	1^a^	0^a^	0^a^	0^a^	0^a^	0^a^	0^a^	0^a^	0^a^	0^a^	0^a^	0^a^	0^a^	0^a^	0^a^	0^a^	0	DnaJ (H sp40) homolog, subfamily B, member 3 [Mm.3075, NM_008299]	Cell defence
GTGCTGGCTTG	**88**	0^a^	0^a^	0^a^	0^a^	0^a^	0^a^	0^a^	0^a^	0^a^	0^a^	0^a^	0^a^	0^a^	0^a^	0^a^	0^a^	0^a^	0^a^	0^a^	0^a^	0^a^		NM	Novel transcript
ACACCCACGCG	**83**	0^a^	0^a^	0^a^	0^a^	0^a^	0^a^	0^a^	0^a^	0^a^	0^a^	0^a^	0^a^	0^a^	0^a^	0^a^	0^a^	0^a^	0^a^	0^a^	0^a^	0^a^	0	sperm atogenic Zip 1 [Mm.23520, NM_030237]	Transcription factor activity
AAAAAGACCAA	**81**	0^a^	0^a^	0^a^	0^a^	0^a^	0^a^	0^a^	0^a^	0^a^	0^a^	0^a^	0^a^	0^a^	0^a^	0^a^	0^a^	0^a^	0^a^	0^a^	0^a^	0^a^	0	centrin 1 [Mm.195831, NM_007593]	Cell structure
AAGGCCTGCCA	**78**	0^a^	0^a^	0^a^	0^a^	0^a^	0^a^	0^a^	0^a^	0^a^	0^a^	0^a^	0^a^	0^a^	0^a^	0^a^	0^a^	0^a^	0^a^	0^a^	0^a^	0^a^	0	chaperonin containing TC P1, subunit 4 [Mm.332809, BU962071]	Cell protein synthesis
AACAATGTTGT	**72**	0^a^	0^a^	0^a^	0^a^	0^a^	0^a^	0^a^	1^a^	0^a^	0^a^	1^a^	0^a^	0^a^	0^a^	0^a^	0^a^	0^a^	0^a^	0^a^	0^a^	0^a^	0	fatty acid binding protein 9, testis [Mm.26654, NM_011598]	Lipid metabolism
TTCAGCAACGG	**72**	0^a^	0^a^	0^a^	0^a^	0^a^	0^a^	0^a^	1^a^	0^a^	0^a^	0^a^	0^a^	0^a^	0^a^	0^a^	0^a^	0^a^	0^a^	0^a^	0^a^	0^a^	0	t-complex protein 10b [Mm.326683, NM_011553]	Cell signaling
TCTCGCAATGG	**63**	0^a^	0^a^	0^a^	0^a^	0^a^	0^a^	1^a^	0^a^	0^a^	0^a^	0^a^	0^a^	0^a^	0^a^	0^a^	0^a^	0^a^	0^a^	0^a^	0^a^	0^a^	0	tubby-like protein 2 [Mm.280778, NM_008807]	Phosphoric diester hydrolase Activity
CTGGATGGTTT	**60**	0^a^	0^a^	0^a^	0^a^	0^a^	0^a^	0^a^	0^a^	0^a^	0^a^	0^a^	0^a^	0^a^	0^a^	0^a^	0^a^	0^a^	0^a^	0^a^	0^a^	0^a^	0	actin-like 7b [Mm.251434, NM_025271]	Cell structure
ACCTGCAGCCT	**58**	0^a^	0^a^	0^a^	0^a^	0^a^	0^a^	0^a^	0^a^	0^a^	0^a^	0^a^	0^a^	0^a^	0^a^	0^a^	0^a^	0^a^	0^a^	0^a^	0^a^	0^a^	0	heat shock protein, alpha-crystallin-related, B9 [Mm.46175, XM_126494]	Cell defence
AACAAAAATCC	**58**	0^a^	0^a^	0^a^	0^a^	0^a^	0^a^	0^a^	0^a^	3	0^a^	0^a^	0^a^	1^a^	1^a^	0^a^	0^a^	0^a^	0^a^	0^a^	0^a^	0^a^	0	testis specific gene A2 [Mm.12743, NM_025290]	Cell signaling
CGCGGAATGCT	**56**	0^a^	0^a^	0^a^	1^a^	0^a^	0^a^	1^a^	1^a^	0^a^	0^a^	0^a^	0^a^	0^a^	1^a^	0^a^	0^a^	0^a^	0^a^	0^a^	0^a^	0^a^	0	PH D finger protein 7 [Mm.5348, BY099053]	Protein synthesis
TCTTGCGGCGG	**55**	0^a^	0^a^	0^a^	0^a^	0^a^	0^a^	0^a^	0^a^	0^a^	1^a^	0^a^	0^a^	0^a^	1^a^	0^a^	0^a^	0^a^	0^a^	0^a^	0^a^	0^a^	0	DnaJ (H sp40) homolog, subfamily B, member 3 [Mm.3075, AK005688]	Cell defence
GAGCAGGTCCA	**48**	0^a^	0^a^	0^a^	0^a^	0^a^	0^a^	0^a^	0^a^	0^a^	0^a^	0^a^	0^a^	0^a^	0^a^	0^a^	0^a^	0^a^	0^a^	0^a^	0^a^	0^a^	0	kinesin family member 2B [Mm.67677, XM_126653]	Cell structure
TCTTCTGCCTC	**46**	1^a^	0^a^	0^a^	1^a^	0^a^	0^a^	0^a^	0^a^	0^a^	0^a^	0^a^	0^a^	0^a^	0^a^	0^a^	0^a^	0^a^	0^a^	0^a^	0^a^	0^a^	0	serine/threonine kinase 22B (sperm iogenesis associated) [Mm.310201, NM_009436]	Metabolism: protein modification
GTGTGCGGACC	**44**	0^a^	0^a^	0^a^	0^a^	0^a^	0^a^	0^a^	2^a^	0^a^	0^a^	0^a^	0^a^	1^a^	0^a^	0^a^	0^a^	0^a^	0^a^	0^a^	0^a^	0^a^	0	sperm associated antigen 4 [Mm.81035, AK028080]	Cell defence
AATCCATCCAG	**44**	0^a^	0^a^	0^a^	0^a^	0^a^	0^a^	0^a^	0^a^	0^a^	0^a^	0^a^	0^a^	1^a^	0^a^	0^a^	0^a^	0^a^	0^a^	0^a^	0^a^	0^a^	0	thioredoxin domain containing 2 (sperm atozoa) [Mm.255732, NM_153519]	Cell defence
GCTCCTTTAAA	**42**	0^a^	0^a^	0^a^	0^a^	0^a^	0^a^	0^a^	0^a^	0^a^	0^a^	0^a^	0^a^	0^a^	0^a^	0^a^	0^a^	0^a^	0^a^	0^a^	0^a^	0^a^	0	cytochrome coxidase, subunit VIIIc [Mm.660, XM_354691]	Energy metabolism
ACCTAGGCAGA	**42**	0	0^a^	0^a^	0^a^	0^a^	0^a^	0^a^	0^a^	0^a^	0^a^	0	0^a^	0^a^	0^a^	0^a^	0^a^	0^a^	0^a^	0^a^	0^a^	0^a^	0	testis specific gene A8 [Mm.143832, AB032764]	Rhabdomere development and photoreceptor cell survival
CTAATCAGGAG	**41**	0^a^	0^a^	0^a^	0^a^	0^a^	0^a^	0^a^	0^a^	0^a^	0^a^	0^a^	0^a^	0^a^	0^a^	0^a^	0^a^	0^a^	0^a^	0^a^	0^a^	0^a^	0	thioredoxin reductase 1 [Mm.210155, NM_025499]	Cell defence
CAAGAGCCTCA	**41**	0^a^	0^a^	0^a^	0^a^	0^a^	0^a^	0^a^	0^a^	0^a^	0^a^	0^a^	0^a^	1^a^	1^a^	0^a^	0^a^	0^a^	0^a^	0^a^	0^a^	0^a^	0	selenoprotein W [Mm.212777, NM_175033]	Cell defence
TGCCCCAACAT	**41**	0^a^	0^a^	0^a^	0^a^	0^a^	0^a^	0^a^	0^a^	0^a^	0^a^	0^a^	0^a^	0^a^	0^a^	0^a^	0^a^	0^a^	0^a^	0^a^	0^a^	0^a^	0	oxysterol binding protein 2 [Mm.348003, BC058602]	Transport
CACGAAGTTCC	**39**	1^a^	0^a^	0^a^	0^a^	0^a^	0^a^	0^a^	1^a^	0^a^	0^a^	0^a^	0^a^	1^a^	0^a^	0^a^	0^a^	0^a^	0^a^	0^a^	0^a^	0^a^	0	neighbor of Brca1 gene 1 [Mm.784, AF227188]	Zing and ion binding
ATAGGATGCTG	**37**	0^a^	0^a^	0^a^	0^a^	0^a^	0^a^	0^a^	0^a^	0^a^	0^a^	0^a^	1^a^	0^a^	0^a^	0^a^	0^a^	0^a^	0^a^	0^a^	0^a^	0^a^	0	tudor domain containing 6 [Mm.329058, NM_198418]	Oogenesis
GCTTGAAGCCT	**35**	0^a^	0^a^	0^a^	0^a^	0^a^	0^a^	0^a^	1^a^	0^a^	0^a^	0^a^	0^a^	0^a^	0^a^	0^a^	0^a^	0^a^	0^a^	0^a^	0^a^	0^a^	0	serine/threonine kinase 22A (sperm iogenesis associated) [Mm.347554, XM_147244]	Metabolism: protein modification
GTGCCTACCTA	**33**	0^a^	0^a^	0^a^	0^a^	0^a^	0^a^	0^a^	0	1^a^	0^a^	0^a^	1^a^	1^a^	2^a^	0^a^	0^a^	0^a^	0^a^	0^a^	0^a^	0^a^	0	Park2 co-regulated [Mm.18889, XM_128418]	Sperm differentiation
TGGGTGAAGGG	**33**	1^a^	0^a^	0^a^	0^a^	0^a^	0^a^	0^a^	0^a^	0^a^	0^a^	0^a^	0^a^	0^a^	0^a^	0^a^	0^a^	0^a^	0^a^	0^a^	0^a^	0^a^	0	tripartite motif protein 17 [Mm.179733, NM_031172]	Ubiquitin-protein ligase activity
GAAATCTTATG	**33**	0^a^	0^a^	0^a^	0^a^	0^a^	0^a^	0^a^	0^a^	0^a^	0^a^	0^a^	0^a^	0^a^	0^a^	0^a^	0^a^	0^a^	0^a^	0^a^	0^a^	0^a^	0	four and a half LIM domains 5 [Mm.87325, NM_021318]	Transcription
GACAAGCAGAC	**32**	0^a^	0^a^	0^a^	0^a^	0^a^	0^a^	1^a^	0^a^	1^a^	0^a^	0^a^	0^a^	1^a^	0^a^	0^a^	0^a^	0^a^	0^a^	0^a^	0^a^	0^a^	0	translin-associated factor X (Tsnax) interacting protein 1 [Mm.28323, NM_024445]	Protein binding
AGGGTGGACAA	**30**	0^a^	0^a^	0^a^	0^a^	0^a^	0^a^	0^a^	0^a^	0^a^	0^a^	0^a^	0^a^	0^a^	0^a^	0^a^	0^a^	0^a^	0^a^	0^a^	0^a^	0^a^	0	DnaJ (H sp40) homolog, subfamily B, member 8 [Mm.272871, NM_019964]	Cell defence
GGCTCAATTTC	**30**	0^a^	0^a^	0^a^	0^a^	0^a^	0^a^	0^a^	0^a^	0^a^	0^a^	0^a^	1^a^	0^a^	1^a^	0^a^	0^a^	0^a^	0^a^	0^a^	0^a^	0^a^	0	StAR -related lipid transfer (STAR T) domain containing 6 [Mm.83623, BC061022]	Cell signaling
TGTTCTTCACT	**30**	0^a^	0^a^	0^a^	0^a^	0^a^	0^a^	0^a^	0^a^	1^a^	0^a^	0^a^	0^a^	0^a^	0^a^	0^a^	0^a^	0^a^	0^a^	0^a^	0^a^	0^a^	0	sperm associated antigen 9 [Mm.260737, AK034670]	Cell defence
CCCTGGGAGAC	**30**	0^a^	2^a^	0^a^	0^a^	0^a^	0^a^	0^a^	1^a^	1	0^a^	1^a^	1^a^	2^a^	0^a^	0^a^	0^a^	0^a^	0^a^	0^a^	0^a^	0^a^	0	sterile alpha motif domain containing 4 [Mm.269139, XM_127686]	Post-transcriptional regulators
GTCGCTGTCTT	**28**	0^a^	0^a^	0^a^	0^a^	0^a^	0^a^	0^a^	0^a^	1^a^	0^a^	0^a^	0^a^	1^a^	0^a^	0^a^	0^a^	0^a^	0^a^	0^a^	0^a^	0^a^	0	fibronectin type 3 and ankyrin repeat domains 1 [Mm.87448, AK017071]	Transcription factor activity and DNA binding
CCTTCTGTCGG	**28**	0^a^	0^a^	0^a^	0^a^	0^a^	0^a^	0^a^	0^a^	0^a^	0^a^	0^a^	0^a^	0^a^	0^a^	0^a^	0^a^	0^a^	0^a^	0^a^	0^a^	0^a^	0	t-complex protein 10b [Mm.326683, NM_009340]	Cell signaling
GCTACGCTCAC	**28**	0^a^	0^a^	0^a^	0^a^	0^a^	0^a^	0^a^	0^a^	0^a^	0^a^	0^a^	0^a^	0^a^	0^a^	0^a^	0^a^	0^a^	0^a^	0^a^	0^a^	0^a^	0	dipeptidase 3 [Mm.173395, NM_027960]	Protein synthesis
CAGACCAACGC	**28**	0^a^	0^a^	0^a^	0^a^	0^a^	0^a^	0^a^	0^a^	0^a^	0^a^	1^a^	0^a^	0^a^	0^a^	0^a^	0^a^	0^a^	0^a^	0^a^	0^a^	0^a^	0	testis specific 10 interacting protein [Mm.329659, AK019010]	Cell signaling
ATGCCTTTCCA	**26**	0^a^	0^a^	0^a^	0^a^	0^a^	0^a^	0^a^	1^a^	0^a^	0^a^	0^a^	0^a^	0^a^	0^a^	0^a^	0^a^	0^a^	0^a^	0^a^	0^a^	0^a^	0	ankyrin repeat domain 5 [Mm.198389, NM_175667]	Cell structure
AACAATATTTA	**26**	0^a^	0^a^	0^a^	1^a^	0^a^	0^a^	1^a^	0^a^	0^a^	1^a^	0^a^	0^a^	0^a^	0^a^	0^a^	0^a^	0^a^	0^a^	0^a^	0^a^	0^a^	0	ubiquitin-conjugating enzyme E2N [Mm.328239, AK005788]	Protein synthesis
GCTGCATAACA	**26**	0^a^	0^a^	0^a^	0^a^	0^a^	0^a^	3	1^a^	0^a^	0^a^	0^a^	1^a^	0^a^	0^a^	0^a^	0^a^	0^a^	0^a^	0^a^	0^a^	0^a^	0	aquaporin 11 [Mm.29756, NM_175105]	Transport
**Prostate**																				0^a^					
ACCCAGACACG	0^a^	**193**	0^a^	0^a^	0^a^	0^a^	0^a^	1^a^	0^a^	0^a^	0^a^	0^a^	0^a^	1^a^	0^a^	0^a^	0^a^	0^a^	0^a^	0^a^	0^a^	0^a^	0	seminal vesicle protein, secretion 2 [Mm.99395, BB872622]	Protein secretion
TGACAAAACGT	0^a^	**99**	0^a^	0^a^	0^a^	0^a^	0^a^	1^a^	0^a^	0^a^	0^a^	0^a^	0^a^	1^a^	0^a^	0^a^	0^a^	0^a^	0^a^	0^a^	0^a^	0^a^	0	beta-microseminoprotein [Mm.2540, NM_020597]	Cell signaling
AAGACGGGTAG	0^a^	**91**	0^a^	0^a^	0^a^	0^a^	0^a^	1^a^	0^a^	0^a^	0^a^	0^a^	0^a^	0^a^	0^a^	0^a^	0^a^	0^a^	0^a^	0^a^	0^a^	0^a^	0	C U B and zona pellucida-like domains 1 [Mm.304207, BE852974]	Cell signaling
GCAACTAGCCT	0^a^	**66**	0^a^	0^a^	0^a^	0^a^	0^a^	1^a^	0^a^	0^a^	0^a^	0^a^	0^a^	0^a^	0^a^	0^a^	0^a^	0^a^	0^a^	0^a^	0^a^	0^a^	0	serine peptidase inhibitor, Kazal type 3 [Mm.272, DV071569]	Peptidase activity
GTGAGAAACAC	0^a^	**52**	0^a^	0^a^	0^a^	0^a^	0^a^	0^a^	0^a^	0^a^	0^a^	0^a^	0^a^	0^a^	0^a^	0^a^	0^a^	0^a^	0^a^	0^a^	0^a^	0^a^		NM	Novel transcript
TATTTTGCAAT	0^a^	**49**	0^a^	0^a^	0^a^	0^a^	0^a^	1^a^	0^a^	0^a^	0^a^	0^a^	0^a^	0^a^	0^a^	0^a^	0^a^	0^a^	0^a^	0^a^	0^a^	0^a^	0	seminal vesicle antigen [Mm.4119, NM_009299]	Protein secretion
CCTGGTGAAAG	0^a^	**45**	0^a^	0^a^	0^a^	0^a^	0^a^	0^a^	0^a^	0^a^	0^a^	0^a^	0^a^	1^a^	0^a^	0^a^	0^a^	0^a^	0^a^	0^a^	0^a^	0^a^	0	mucin 10, submandibular gland salivary mucin [Mm.200411, NM_008644]	Spermatogenesis

^a ^Significant difference vs. no. of tags show n in bold (p < 0,05)

**Table 3:** Specific genes expressed in the female sexual organs

																Tissues from GEO and accession number									
																			
Tag	Testis	Prostate	**Ovary**	**Mammary gland**	**Uterus**	**Vagina**	Skin	Liver	Adipose tissue	Lung	Bone	Skeletal muscle	**Cerebral cortex**	**Hypothalamus**	**Pituitary gland**	Mouse embryonic fibroblasts GSM7759	Heart normal GSM106587	Thymus normal GSM106596	Bladder normal GSM106599	Kidney normal GSM56242	Spleen normal GSM106591	Pancreas Normal GSM106592	Intestine UniGene and EST expression (%)	Description [UniGene cluster, GenBank Accession No]	General function
**Ovary**																									
TGGCAGAAGCC	0^a^	0^a^	**73**	0^a^	0^a^	0^a^	0^a^	0^a^	0^a^	0^a^	0^a^	0^a^	0^a^	0^a^	0^a^	0^a^	0^a^	0^a^	0^a^	0^a^	0^a^	0^a^	0	RIKEN cDNA 4930583H14 gene [Mm.273339, NM_026358]	Protein secretion
GTCAACACAGG	2^a^	0^a^	**38**	0^a^	1^a^	0^a^	0^a^	0^a^	0^a^	0^a^	0^a^	0^a^	0^a^	0^a^	0^a^	0^a^	0^a^	0^a^	0^a^	0^a^	0^a^	0^a^		NM	Novel transcript
CATATGTTGAT	2^a^	0^a^	**36**	0^a^	0^a^	0^a^	0^a^	0^a^	0^a^	0^a^	0^a^	0^a^	0^a^	1^a^	0^a^	0^a^	0^a^	0^a^	0^a^	0^a^	0^a^	0^a^	0	RIKEN cDNA 4921521F21 gene [Mm.18725, NM_027582]	catalyzes the reduction of 2,5-diketo-d-gluconic acid
**Mammary gland**																									
TCCGGAGAAAA	0^a^	1^a^	0^a^	**35**	0^a^	0^a^	1^a^	0^a^	1^a^	0^a^	0^a^	0^a^	0^a^	0^a^	0^a^	0^a^	0^a^	0^a^	0^a^	0^a^	0^a^	0^a^		NM	Novel transcript
CTAGGTGGTGC	0^a^	0^a^	0^a^	**32**	1^a^	0^a^	0^a^	1^a^	0^a^	0^a^	0^a^	0^a^	0^a^	0^a^	0^a^	0^a^	0^a^	0^a^	0^a^	0^a^	0^a^	0^a^	0	glycosylation dependent cell adhesion molecule 1 [Mm.219621, NM_008134]	Cell signaling
**Uterus**																									
TCTGACGATGT	0^a^	0^a^	0^a^	0^a^	**130**	0^a^	0^a^	0^a^	0^a^	0^a^	0^a^	0^a^	0^a^	0^a^	0^a^	0^a^	0^a^	0^a^	0^a^	0^a^	0^a^	1^a^	73	proline-rich acidic protein 1 [Mm.141646, NM_009475]	Cell signaling
GAAGCTGTATG	0^a^	0^a^	2^a^	0^a^	**33**	0^a^	0^a^	0^a^	0^a^	0^a^	0^a^	0^a^	0^a^	0^a^	0^a^	0^a^	1^a^	0^a^	0^a^	2^a^	0^a^	0^a^	25	Hydroxysteroid 11-beta dehydrogenase 2 [Mm.5079, BC014753]	Steroid hormone synthesis
TACATAGATGG	0^a^	1^a^	0^a^	0^a^	**31**	0^a^	1^a^	1^a^	0^a^	0^a^	0^a^	0^a^	0^a^	0^a^	0^a^	0^a^	0^a^	0^a^	0^a^	0^a^	0^a^	0^a^	85	chloride channel calcium activated 3 [Mm.33483, NM_017474]	Cell signaling
**Vagina**																									
CTGTATTTGGG	0^a^	0^a^	0^a^	0^a^	2^a^	**114**	0^a^	0^a^	0^a^	0^a^	0^a^	0^a^	0^a^	0^a^	0^a^	0^a^	0^a^	0^a^	0^a^	0^a^	0^a^	0^a^		NM	Novel transcript
AAGCACCAAAT	0^a^	0^a^	0^a^	0^a^	2^a^	**85**	0^a^	0^a^	0^a^	0^a^	0^a^	0^a^	0^a^	0^a^	0^a^	0^a^	0^a^	0^a^	0^a^	0^a^	0^a^	0^a^		NM	Novel transcript
AAAGCATCCTT	0^a^	0^a^	0^a^	0^a^	1^a^	**77**	0^a^	0^a^	0^a^	0^a^	0^a^	0^a^	0^a^	0^a^	0^a^	0^a^	0^a^	0^a^	0^a^	0^a^	0^a^	0^a^	0	keratin intermediate filament 16a [Mm.343031, XM_484082]	Cell structure
CAGAACCTCAA	0^a^	0^a^	0^a^	0^a^	1^a^	**39**	0^a^	0^a^	0^a^	0^a^	0^a^	0^a^	0^a^	1^a^	0^a^	0^a^	0^a^	0^a^	0^a^	0^a^	0^a^	0^a^	26	keratin complex 1, acidic, gene 13 [Mm.4646, BY709670]	Cell structure
GCCTTGGAGGT	0^a^	0^a^	0^a^	0^a^	2^a^	**35**	0^a^	0^a^	0^a^	0^a^	0^a^	0^a^	0^a^	0^a^	0^a^	0^a^	0^a^	0^a^	0^a^	0^a^	0^a^	0^a^	0	keratin complex 2, basic, gene 6 g [Mm.22657, NM_010669]	Cell structure
GGGACTCCTCC	0^a^	0^a^	0^a^	0^a^	0^a^	**34**	1^a^	1^a^	0^a^	0^a^	0^a^	0^a^	0^a^	0^a^	0^a^	0^a^	0^a^	0^a^	0^a^	0^a^	0^a^	9^a^	84	retinol binding protein 2, cellular [Mm.12825, NM_009034]	Intracellular transport

^a ^Significant difference vs. no. of tags show n in bold (p < 0,05)

**Table 4:** Specific genes expressed in the skin, liver, adipose tissue, lungs, bone, and skeletal muscle

																Tissues from GEO and accession number									
																			
Tag	**Testis**	**Prostate**	Ovary	Mammary gland	Uterus	Vagina	**Skin**	**Liver**	Adipose tissue	Lung	Bone	Skeletal muscle	Cerebral cortex	Hypothalamus	Pituitary gland	Mouse embryonic fibroblasts GSM7759	Heart normal GSM106587	Thymus normal GSM106587	Bladder normal GSM106599	Kidney normal GSM56242	Spleen normal GSM106591	Pancreas normal GSM 106592	Intestine UniGene and EST expression (%)	Description [UniGene cluster, GenBank Accession No]	General function
**Skin**																									
ATTCCCTGTTA	0^a^	0^a^	0^a^	0^a^	0^a^	0^a^	**299**	2^a^	0^a^	0^a^	0^a^	0^a^	0^a^	0^a^	0^a^	0^a^	0^a^	0^a^	0^a^	0^a^	0^a^	0^a^	0	keratin associated protein 8-1 [Mm.13979, D86423]	Cell structure
AAGTGAAAGCA	0^a^	0^a^	0^a^	0^a^	0^a^	0^a^	**204**	2^a^	0^a^	0^a^	0^a^	0^a^	0^a^	0^a^	0^a^	0^a^	0^a^	0^a^	0^a^	0^a^	0^a^	0^a^	0	trichohyalin – human [Mm.1160, CD545918]	Fibrillar forming collagen
CCTCCATTTCC	0^a^	0^a^	0^a^	0^a^	0^a^	0^a^	**86**	0^a^	0^a^	0^a^	0^a^	0^a^	0^a^	0^a^	0^a^	0^a^	0^a^	0^a^	0^a^	0^a^	0^a^	0^a^	0	keratin complex 1, acidic, gene 4 [Mm.289644, NM_027563]	Cell structure
GTTCTCAGTAT	0^a^	0^a^	0^a^	0^a^	0^a^	0^a^	**66**	1^a^	0^a^	0^a^	0^a^	0^a^	0^a^	0^a^	0^a^	0^a^	0^a^	0^a^	0^a^	0^a^	0^a^	0^a^	0	keratin associated protein 6-1 [Mm.310892, D86419]	Cell structure
TTGCTTCTGGG	0^a^	0^a^	0^a^	0^a^	0^a^	0^a^	**65**	0^a^	0^a^	0^a^	0^a^	0^a^	0^a^	0^a^	0^a^	0^a^	0^a^	0^a^	0^a^	0^a^	0^a^	0^a^	0	keratin associated protein 3-2 [Mm.46389, NM_025720]	Cell structure
AAGCTTTGATA	0^a^	0^a^	0^a^	0^a^	0^a^	0^a^	**59**	0^a^	0^a^	0^a^	0^a^	0^a^	0^a^	0^a^	0^a^	0^a^	0^a^	0^a^	0^a^	0^a^	0^a^	0^a^	0	keratin associated protein 16-5 [Mm.28425, NM_130857]	Cell structure
GCTTCACCTTG	0^a^	0^a^	0^a^	0^a^	0^a^	0^a^	**40**	0^a^	0^a^	0^a^	0^a^	0^a^	0^a^	0^a^	0^a^	0^a^	0^a^	0^a^	0^a^	0^a^	0^a^	0^a^	0	keratin associated protein 11-1 [Mm.310906, AV018433]	Cell structure
ATGGTCTGAGC	0^a^	0^a^	0^a^	0^a^	0^a^	0^a^	**40**	0^a^	0^a^	0^a^	0^a^	0^a^	0^a^	0^a^	0^a^	0^a^	0^a^	0^a^	0^a^	0^a^	0^a^	0^a^	0	S100 calcium binding protein A17 [Mm.68064, NM_027762]	Cell signaling
CAACTCCTTTG	0^a^	0^a^	0^a^	0^a^	0^a^	0^a^	**35**	0^a^	0^a^	0^a^	0^a^	0^a^	0^a^	0^a^	0^a^	0^a^	0^a^	0^a^	0^a^	0^a^	0^a^	0^a^	0	keratin complex 1, acidic, gene 1 [Mm.19109, NM_010659]	Cell structure
GGCCTGGCTTA	0^a^	0^a^	0^a^	0^a^	0^a^	0^a^	**35**	1^a^	1^a^	0^a^	0^a^	0^a^	0^a^	0^a^	0^a^	0^a^	0^a^	0^a^	0^a^	0^a^	0^a^	0^a^	0	lymphocyte antigen 6 complex, locus G6C [Mm.215096, NM_023463]	Cell defence
GTACTGTCTTG	0^a^	0^a^	0^a^	0^a^	0^a^	0^a^	**34**	0^a^	0^a^	0^a^	0^a^	0^a^	0^a^	0^a^	0^a^	0^a^	0^a^	0^a^	0^a^	0^a^	0^a^	0^a^	0	keratin associated protein 4.7 (Homo sapiens) [Mm.340791, AV089658]	Cell structure
TCCTGCACAAT	0^a^	0^a^	0^a^	0^a^	0^a^	0^a^	**25**	1^a^	0^a^	0^a^	0^a^	0^a^	0^a^	0^a^	0^a^	0^a^	0^a^	0^a^	0^a^	0^a^	0^a^	0^a^	0	G protein-coupled receptor, family C, group 5, member D [Mm.49902, NM_053118]	Cell signaling
**Liver**																									
AAGACTCAGGA	0^a^	0^a^	0^a^	0^a^	0^a^	0^a^	0^a^	**185**	0^a^	0^a^	3^a^	0^a^	0^a^	0^a^	0^a^	0^a^	0^a^	0^a^	0^a^	0^a^	0^a^	0^a^	0	albumin 1 [Mm.16773, NM_009654]	Cell defence
TCGGACCATAG	0^a^	0^a^	0^a^	0^a^	0^a^	0^a^	0^a^	**120**	1^a^	0^a^	1^a^	0^a^	0^a^	1^a^	0^a^	0^a^	0^a^	0^a^	0^a^	0^a^	0^a^	0^a^	0	alpha 1 microglobulin/bikunin [Mm.2197, NM_007443]	Cell defence
CAAATAGGTTG	0^a^	0^a^	0^a^	0^a^	0^a^	0^a^	0^a^	**101**	0^a^	0^a^	0^a^	0^a^	0^a^	0^a^	0^a^	0^a^	0^a^	0^a^	0^a^	0^a^	0^a^	0^a^	0	serine (or cysteine) proteinase inhibitor, clade A, member 1a [Mm.259233, AI527352]	Cell defence
ACCCTTAGAGA	0^a^	0^a^	0^a^	0^a^	0^a^	0^a^	0^a^	**93**	0^a^	0^a^	0^a^	0^a^	0^a^	0^a^	0^a^	0^a^	0^a^	0^a^	0^a^	0^a^	0^a^	0^a^	0	betaine-homocysteine methyltransferase [Mm.329582, NM_016668]	Amino acid metabolism
GCCACGCCCCC	0^a^	0^a^	0^a^	0^a^	0^a^	0^a^	0^a^	**89**	1^a^	0^a^	1^a^	0^a^	0^a^	0^a^	0^a^	0^a^	0^a^	0^a^	0^a^	0^a^	0^a^	0^a^	0	4-hydroxyphenylpyruvic acid dioxygenase [Mm.6584, NM_008277]	Amino acid metabolism
GTGATTGCTGA	0^a^	1^a^	0^a^	0^a^	0^a^	0^a^	0^a^	**79**	0^a^	0^a^	0^a^	1^a^	0^a^	0^a^	0^a^	0^a^	0^a^	0^a^	0^a^	0^a^	0^a^	0^a^	0	murinoglobulin 2 [Mm.244937, NM_008646]	Cell defence
TGTTCCGTCTG	0^a^	0^a^	0^a^	0^a^	0^a^	0^a^	0^a^	**67**	0^a^	0^a^	1^a^	0^a^	0^a^	0^a^	0^a^	0^a^	0^a^	0^a^	0^a^	0^a^	0^a^	0^a^	0	coagulation factor II [Mm.89048, NM_010168]	Cell defence
TTTCTTAAATC	0^a^	0^a^	0^a^	0^a^	0^a^	0^a^	0^a^	**67**	0^a^	0^a^	0^a^	0^a^	0^a^	0^a^	0^a^	0^a^	0^a^	0^a^	0^a^	0^a^	0^a^	0^a^	0	esterase 31-like [Mm.347422, NM_144511]	Cell defence
TTGTCCTCGTA	0^a^	0^a^	0^a^	0^a^	0^a^	0^a^	0^a^	**61**	0^a^	2^a^	0^a^	1^a^	0^a^	0^a^	0^a^	0^a^	0^a^	0^a^	0^a^	0^a^	0^a^	0^a^	0	inter alpha-trypsin inhibitor, heavy chain 4 [Mm.211681, NM_018746]	Cell defence
GCGATGAAATC	0^a^	0^a^	0^a^	0^a^	0^a^	0^a^	0^a^	**59**	0^a^	0^a^	0^a^	1^a^	0^a^	0^a^	0^a^	0^a^	0^a^	0^a^	0^a^	0^a^	0^a^	0^a^	0	esterase 1 [Mm.88078, AI256598]	Cell defence
CTCATCGTATG	0^a^	0^a^	0^a^	0^a^	0^a^	0^a^	0^a^	**57**	0^a^	0^a^	0^a^	0^a^	0^a^	0^a^	0^a^	0^a^	0^a^	0^a^	0^a^	0^a^	0^a^	0^a^		NM	Novel transcript
GGCACCTTCAC	0^a^	0^a^	0^a^	0^a^	0^a^	0^a^	0^a^	**56**	1^a^	0^a^	1^a^	0^a^	0^a^	0^a^	0^a^	0^a^	0^a^	0^a^	0^a^	0^a^	0^a^	0^a^	0	peptidoglycan recognition protein 4 [Mm.316644, XM_357978]	Cell defence
GAGCTGTTTCT	0^a^	0^a^	0^a^	0^a^	0^a^	0^a^	0^a^	**56**	0^a^	0^a^	0^a^	1^a^	0^a^	0^a^	0^a^	0^a^	0^a^	0^a^	0^a^	0^a^	0^a^	0^a^	0	cytochrome P450, family 2, subfamily c, polypeptide 40 [Mm.335940, NM_010004]	Steroid hormone synthesis
TTGCAAGGCTG	0^a^	0^a^	0^a^	0^a^	0^a^	0^a^	0^a^	**46**	1^a^	1^a^	0^a^	0^a^	0^a^	0^a^	0^a^	0^a^	0^a^	0^a^	0^a^	0^a^	1^a^	0^a^	0	albumin 1 [Mm.16773, AI747247]	Cell defence
GAGCTCTTCCT	2^a^	0^a^	0^a^	0^a^	0^a^	0^a^	0^a^	**45**	0^a^	0^a^	0^a^	1^a^	0^a^	0^a^	0^a^	0^a^	0^a^	0^a^	0^a^	0^a^	0^a^	0^a^	0	cytochrome P450, family 2, subfamily d, polypeptide 26 [Mm.29064, NM_029562]	Steroid hormone synthesis
AGACCTTGGGA	0^a^	0^a^	0^a^	0^a^	0^a^	0^a^	0^a^	**44**	1^a^	0^a^	0^a^	0^a^	0^a^	0^a^	0^a^	0^a^	0^a^	0^a^	0^a^	0^a^	0^a^	0^a^	0	kininogen 2 [Mm.2160, NM_023125]	Cell defence
GTTGCTGACCG	0^a^	0^a^	0^a^	0^a^	0^a^	0^a^	0^a^	**43**	0^a^	0^a^	0^a^	0^a^	0^a^	0^a^	0^a^	0^a^	0^a^	0^a^	0^a^	0^a^	0^a^	0^a^	0	plasminogen [Mm.971, NM_008877]	Protein synthesis
AAAACAGAAAA	1^a^	0^a^	0^a^	0^a^	0^a^	0^a^	0^a^	**39**	0^a^	0^a^	0^a^	0^a^	0^a^	0^a^	0^a^	0^a^	0^a^	0^a^	0^a^	0^a^	0^a^	0^a^		NM	Novel transcript
TGTGTTATTTT	0^a^	0^a^	0^a^	0^a^	0^a^	0^a^	0^a^	**35**	0^a^	0^a^	1^a^	0^a^	0^a^	0^a^	0^a^	0^a^	0^a^	0^a^	0^a^	0^a^	0^a^	0^a^	0	regucalcin [Mm.2118, NM_009060]	Regulation of enzymatic activity
AAAGTCCTCGA	0^a^	0^a^	0^a^	0^a^	0^a^	0^a^	0^a^	**34**	0^a^	0^a^	0^a^	0^a^	0^a^	0^a^	0^a^	0^a^	0^a^	0^a^	0^a^	0^a^	0^a^	0^a^	0	aldo-keto reductase family 1, member C6 [Mm.196666, NM_030611]	Catalytic activity
GATACAGACTA	0^a^	0^a^	0^a^	0^a^	0^a^	0^a^	0^a^	**33**	0^a^	0^a^	0^a^	0^a^	0^a^	0^a^	0^a^	0^a^	0^a^	0^a^	0^a^	0^a^	0^a^	0^a^		NM	Novel transcript
GACACACACTA	0^a^	0^a^	0^a^	0^a^	0^a^	0^a^	0^a^	**32**	0^a^	1^a^	1^a^	0^a^	0^a^	0^a^	0^a^	0^a^	0^a^	0^a^	0^a^	0^a^	0^a^	0^a^		NM	Novel transcript
ACATTTCCAGA	0^a^	0^a^	0^a^	0^a^	1^a^	0^a^	0^a^	**28**	0^a^	0^a^	0^a^	0^a^	0^a^	0^a^	0^a^	0^a^	0^a^	0^a^	0^a^	2^a^	0^a^	0^a^	0	silica-induced gene 111 [Mm.330825, AV058272]	promoting cell survival
GGGACATTCGG	0^a^	1^a^	0^a^	0^a^	1^a^	0^a^	0^a^	**25**	0^a^	1^a^	0^a^	1^a^	0^a^	0^a^	0^a^	0^a^	0^a^	0^a^	0^a^	0^a^	0^a^	0^a^	0	solute carrier family 27 (fatty acid transporter), member 2 [Mm.290044, BC013442]	Transport
AAATTATTCCT	0^a^	0^a^	0^a^	0^a^	0^a^	0^a^	0^a^	**24**	0^a^	0^a^	0^a^	0^a^	0^a^	0^a^	0^a^	0^a^	0^a^	0^a^	0^a^	0^a^	0^a^	0^a^	0	major urinary protein 1 [Mm.335875, AW110035]	Cell defence
**Adipose tissue**																									
CCAGCACTCAA	0^a^	0^a^	0^a^	0^a^	0^a^	0^a^	1^a^	0^a^	**31**	1^a^	0^a^	0^a^	0^a^	0^a^	0^a^	0^a^	0^a^	0^a^	0^a^	0^a^	0^a^	0^a^	0	leptin [Mm.277072, NM_008493]	Cell signaling
TACCTTTCATA	0^a^	1^a^	0^a^	0^a^	1^a^	0^a^	0^a^	0^a^	**29**	0^a^	0^a^	0^a^	0^a^	0^a^	0^a^	0^a^	0^a^	0^a^	0^a^	0^a^	0^a^	0^a^	7	lectin, galactose binding, soluble 12 [Mm.298242, NM_019516]	Cell division
**Lung**																									
CAAGGATCTAC	0^a^	0^a^	0^a^	0^a^	0^a^	0^a^	0^a^	1^a^	0^a^	**85**	0^a^	0^a^	0^a^	0^a^	0^a^	0^a^	0^a^	0^a^	0^a^	0^a^	0^a^	0^a^	0	surfactant associated protein D [Mm.1321, NM_009160]	Cell defence
TGTCTGCCTCT	0^a^	1^a^	0^a^	0^a^	0^a^	0^a^	1^a^	1^a^	1^a^	**45**	0^a^	0^a^	0^a^	0^a^	0^a^	0^a^	0^a^	0^a^	0^a^	0^a^	0^a^	0^a^	0	claudin 18 [Mm.35090, AK033657]	Cell signaling
ACCCACACTCC	0^a^	0^a^	0^a^	0^a^	0^a^	0^a^	0^a^	1^a^	0^a^	**40**	0^a^	0^a^	0^a^	0^a^	0^a^	0^a^	0^a^	0^a^	0^a^	0^a^	0^a^	0^a^	0	surfactant associated protein A1 [Mm.46062, NM_023134]	Cell defence
GCTCACAGAAA	0^a^	1^a^	0^a^	0^a^	0^a^	0^a^	0^a^	1^a^	0^a^	**35**	0^a^	0^a^	0^a^	1^a^	1^a^	0^a^	0^a^	0^a^	0^a^	0^a^	0^a^	0^a^	0	palate, lung, and nasal epithelium carcinoma associated [Mm.268852, NM_011126]	Cell defence
GGCATCCCATT	0^a^	0^a^	0^a^	0^a^	0^a^	0^a^	0^a^	0^a^	0^a^	**31**	0^a^	1^a^	0^a^	0^a^	0^a^	0^a^	0^a^	0^a^	0^a^	0^a^	0^a^	0^a^	0	secretoglobin, family 3A, member 1 [Mm.22802, NM_170727]	Cell defence
**Bone**																									
CTTTCCTGGGT	0^a^	0^a^	0^a^	0^a^	0^a^	0^a^	0^a^	1^a^	0^a^	0^a^	**144**	0^a^	0^a^	0^a^	0^a^	0^a^	0^a^	0^a^	0^a^	0^a^	0^a^	0^a^	0	proteoglycan 2, bone marrow [Mm.142727, NM_008920]	Cell defence
TTTTGGGCACA	0^a^	0^a^	0^a^	0^a^	0^a^	0^a^	1^a^	1^a^	0^a^	0	**122**	0^a^	0^a^	0^a^	0^a^	0^a^	4^a^	0^a^	0^a^	0^a^	0^a^	0^a^	0	solute carrier family 4 (anion exchanger), member 1 [Mm.7248, NM_011403]	transport
TATGTGGACTG	0^a^	0^a^	0^a^	0^a^	0^a^	0^a^	1^a^	2^a^	1^a^	0^a^	**59**	0^a^	1^a^	1^a^	0^a^	0^a^	1^a^	0^a^	0^a^	0^a^	0^a^	0^a^	0	proteinase 3 [Mm.2364, NM_011178]	Cell defence
TGCCGCCGTCG	0^a^	0^a^	0^a^	0^a^	0^a^	0^a^	1^a^	1^a^	0^a^	0^a^	**52**	0^a^	0^a^	0^a^	0^a^	0^a^	0^a^	0^a^	0^a^	0^a^	0^a^	0^a^	0	neutrophil elastase [Mm.262194, BX632826]	Cell defence
ACAGGAAGAGC	0^a^	0^a^	0^a^	0^a^	0^a^	0^a^	0^a^	0^a^	0^a^	0^a^	**39**	0^a^	0^a^	1^a^	0^a^	0^a^	0^a^	0^a^	0^a^	0^a^	0^a^	0^a^	0	eosinophil peroxidase [Mm.1315, NM_007946]	Cell defence
CCCTGGATCAA	0^a^	0^a^	0^a^	0^a^	0^a^	0^a^	0^a^	0^a^	0^a^	0^a^	**29**	0^a^	0^a^	0^a^	0^a^	0^a^	0^a^	0^a^	0^a^	0^a^	0^a^	0^a^	0	cathepsin G [Mm.4858, NM_007800]	Cell defence
CTGAGGTGGGC	0^a^	0^a^	0^a^	0^a^	0^a^	0^a^	0^a^	1^a^	0^a^	0^a^	**25**	0^a^	0^a^	0^a^	0^a^	0^a^	0^a^	0^a^	0^a^	0^a^	0^a^	0^a^	0	claudin 13 [Mm.86652, NM_020504]	Cell signaling
GAAATATTCAC	0^a^	0^a^	0^a^	0^a^	0^a^	0^a^	0^a^	0^a^	0^a^	1^a^	**25**	0^a^	0^a^	0^a^	0^a^	0^a^	0^a^	0^a^	0^a^	0^a^	0^a^	0^a^	0	membrane-spanning 4-domains, subfamily A, member 3 [Mm.119630, NM_133246]	Cell defence
AAAGTGTGACC	0^a^	0^a^	0^a^	0^a^	0^a^	0^a^	0^a^	1^a^	0^a^	0^a^	**25**	1^a^	0^a^	0^a^	0^a^	0^a^	0^a^	0^a^	0^a^	0^a^	0^a^	0^a^	72	carbonic anhydrase 1 [Mm.273195, NM_009799]	Cell defence
**Skeletal muscle**																									
TGGAACGAGCA	0^a^	1^a^	0^a^	0^a^	0^a^	0^a^	1^a^	0^a^	0^a^	0^a^	1^a^	**68**	0^a^	0^a^	0^a^	0^a^	0^a^	0^a^	0^a^	0^a^	0^a^	0^a^		NM	Novel transcript
TGGGCAGCCTT	0^a^	0^a^	0^a^	0^a^	0^a^	0^a^	2^a^	0^a^	0^a^	0^a^	1^a^	**58**	0^a^	0^a^	0^a^	0^a^	0^a^	0^a^	0^a^	0^a^	0^a^	0^a^	0	myosin light chain, phosphorylatable, fast skeletal muscle [Mm.14526, AV165375]	Cell structure
GACCTGAGGGC	0^a^	0^a^	0^a^	0^a^	0^a^	0^a^	1^a^	1^a^	0^a^	0^a^	0^a^	**41**	0^a^	0^a^	0^a^	0^a^	0^a^	0^a^	0^a^	0^a^	0^a^	0^a^		NM	Novel transcript
ACCTCTATATA	0^a^	0^a^	0^a^	2^a^	0^a^	0^a^	1^a^	0^a^	0^a^	1^a^	0^a^	**38**	0^a^	0^a^	0^a^	0^a^	0^a^	0^a^	0^a^	0^a^	0^a^	0^a^	0	tropomyosin 3, gamma [Mm.240839, U04541]	Cell structure

^a ^Significant difference vs. no. of tags shown in bold (p < 0,05)

**Table 5:** Specific genes expressed in the brain

																Tissues from GEO and accession number									
																			
Tag	Testis	Prostate	**Ovary**	**Mammary gland**	**Uterus**	**Vagina**	Skin	Liver	Adipose tissue	Lung	Bone	Skeletal muscle	**Cerebral cortex**	**Hypothalamus**	**Pituitary gland**	Mouse embryonic fibroblasts GSM7759	Heart normal GSM106587	Thymus normal GSM106596	Bladder normal GSM106599	Kidney normal GSM56242	Spleen normal GSM106591	Pancreas Normal GSM106592	Intestine UniGene and EST expression (%)	Description [UniGene cluster, GenBank Accession No]	General function
**Cerebral cortex**																									
GTCGTCCTCTA	2^a^	0^a^	0^a^	0^a^	0^a^	0^a^	0^a^	0^a^	0^a^	1^a^	0^a^	0^a^	**47**	0^a^	0^a^	0^a^	1^a^	0^a^	0^a^	0^a^	0^a^	0^a^	0	solute carrier family 17 (sodium-dependent inorganic phosphate cotransporter), member 7 [Mm.255631, NM_182993]	Transport
TGACCTTGGCC	0^a^	0^a^	0^a^	0^a^	0^a^	0^a^	0^a^	0^a^	0^a^	0^a^	0^a^	0^a^	**41**	1^a^	0^a^	0^a^	0^a^	0^a^	0^a^	0^a^	0^a^	0^a^	0	gene model 748, (NCBI) [Mm.102203, AK122363]	Protein binding and heme Binding
GTACTTGGCTG	0^a^	0^a^	0^a^	0^a^	0^a^	0^a^	0^a^	0^a^	0^a^	0^a^	0^a^	0^a^	**24**	1^a^	2^a^	0^a^	0^a^	0^a^	0^a^	0^a^	0^a^	0^a^	0	synaptic vesicle glycoprotein 2 b [Mm.273082, NM_153579]	Transporter activity
**Hypothalamus**																									
TTGGCAAGTCT	0^a^	0^a^	0^a^	0^a^	0^a^	0^a^	0^a^	0^a^	0^a^	0^a^	0^a^	0^a^	0^a^	**198**	0^a^	0^a^	0^a^	0^a^	0^a^	0^a^	0^a^	0^a^	0	RIKEN cDNA A230109K 23 gene [Mm.200361, BC048534]	neurotransmitter or neuromodulator
TGTGACGCTGG	0^a^	0^a^	0^a^	0^a^	0^a^	0^a^	0^a^	1^a^	0^a^	0^a^	0^a^	0^a^	1^a^	**37**	0^a^	0^a^	0^a^	0^a^	0^a^	0^a^	0^a^	0^a^	0	cocaine and amphetamine regulated transcript [Mm.75498, NM_013732]	Cell signaling
AGTTCCTTCGC	0^a^	1^a^	0^a^	0^a^	0^a^	0^a^	0^a^	0^a^	0^a^	0^a^	0^a^	0^a^	1^a^	**34**	0^a^	0^a^	0^a^	0^a^	0^a^	0^a^	0^a^	0^a^	0	calbindin 2 [Mm.2755, NM_007586]	Calciunion binding
GGCCGCCGCGC	0^a^	0^a^	0^a^	0^a^	0^a^	0^a^	0^a^	0^a^	0^a^	0^a^	0^a^	0^a^	1^a^	**33**	0^a^	0^a^	0^a^	0^a^	0^a^	0^a^	0^a^	0^a^	0	hypocretin [Mm.10096, NM_010410]	Cell signaling
**Pituitary gland**																									
AAGTGTCGCCG	0^a^	0^a^	0^a^	0^a^	0^a^	0^a^	0^a^	0^a^	0^a^	0^a^	0^a^	0^a^	0^a^	0^a^	**14963**	0^a^	0^a^	0^a^	0^a^	0^a^	0^a^	0^a^	0	growth horm one [Mm.343934, NM_008117]	Cell signaling
GGCGAGCTGAT	0^a^	0^a^	0^a^	0^a^	1^a^	0^a^	0^a^	1^a^	0^a^	0^a^	0^a^	0^a^	0^a^	0^a^	**1496**	0^a^	1^a^	0^a^	0^a^	2^a^	0^a^	0^a^	0	Pro-opiomelanocortin-alpha [Mm.277996, NM_008895]	Cell signaling
GCGGGAAAAGC	0^a^	0^a^	0^a^	0^a^	0^a^	0^a^	0^a^	0^a^	0^a^	0^a^	0^a^	0^a^	0^a^	1^a^	**1383**	0^a^	0^a^	0^a^	0^a^	0^a^	0^a^	0^a^		NM	Novel transcript
CTTGGGTGCAA	0^a^	0^a^	0^a^	0^a^	0^a^	0^a^	0^a^	0^a^	0^a^	0^a^	0^a^	0^a^	0^a^	0^a^	**1307**	0^a^	0^a^	0^a^	0^a^	0^a^	0^a^	0^a^		prolactin [Mm.1270, NM_011164]	Cell signaling
AAGTGTCGCCT	0^a^	0^a^	0^a^	0^a^	0^a^	0^a^	0^a^	0^a^	0^a^	0^a^	0^a^	0^a^	0^a^	0^a^	**601**	0^a^	0^a^	0^a^	0^a^	0^a^	0^a^	0^a^		NM	Novel transcript
TTGGCGTCAAA	0^a^	0^a^	0^a^	0^a^	0^a^	0^a^	0^a^	0^a^	0^a^	0^a^	0^a^	0^a^	0^a^	0^a^	**436**	0^a^	0^a^	0^a^	0^a^	0^a^	0^a^	0^a^		NM	Novel transcript
AAGTGTCGCCA	0^a^	0^a^	0^a^	0^a^	0^a^	0^a^	0^a^	0^a^	0^a^	0^a^	0^a^	0^a^	0^a^	0^a^	**408**	0^a^	0^a^	0^a^	0^a^	0^a^	0^a^	0^a^		NM	Novel transcript
TCGGTTCTCTG	0^a^	0^a^	0^a^	0^a^	0^a^	0^a^	0^a^	0^a^	0^a^	0^a^	0^a^	0^a^	0^a^	0^a^	**329**	0^a^	0^a^	0^a^	0^a^	0^a^	0^a^	0^a^		NM	Novel transcript
ACGTACTTCCG	0^a^	0^a^	0^a^	0^a^	0^a^	0^a^	0^a^	0^a^	0^a^	0^a^	0^a^	0^a^	0^a^	1^a^	**222**	0^a^	0^a^	0^a^	0^a^	0^a^	0^a^	0^a^		NM	Novel transcript
CGCAGCGACGA	0^a^	0^a^	0^a^	0^a^	0^a^	0^a^	0^a^	0^a^	0^a^	0^a^	0^a^	0^a^	0^a^	0^a^	**218**	0^a^	0^a^	0^a^	0^a^	0^a^	0^a^	0^a^		NM	Novel transcript
GCTGGGGCCCG	0^a^	0^a^	0^a^	0^a^	0^a^	0^a^	0^a^	0^a^	0^a^	0^a^	0^a^	0^a^	0^a^	0^a^	**194**	0^a^	0^a^	0^a^	0^a^	0^a^	0^a^	0^a^		NM	Novel transcript
ACCCGCAGGTA	0^a^	0^a^	0^a^	0^a^	0^a^	0^a^	0^a^	0^a^	0^a^	0^a^	0^a^	0^a^	0^a^	0^a^	**114**	0^a^	0^a^	0^a^	0^a^	0^a^	0^a^	0^a^		NM	Novel transcript
GTCCGAGTACT	2^a^	0^a^	0^a^	0^a^	0^a^	0^a^	0^a^	0^a^	0^a^	1^a^	0^a^	0^a^	0^a^	0^a^	**75**	0^a^	0^a^	0^a^	0^a^	0^a^	0^a^	0^a^	0	luteinizing hormone beta [Mm.57061, NM_008497]	Cell signaling
TTACTCCTTAT	0^a^	0^a^	0^a^	0^a^	0^a^	0^a^	0^a^	0^a^	0^a^	0^a^	0^a^	0^a^	0^a^	0^a^	**49**	0^a^	0^a^	0^a^	0^a^	0^a^	0^a^	0^a^	0	thyroid stimulating hormone, beta subunit [Mm.110730, NM_009432]	Thyroid structure and metabolism

^a ^Significant difference vs. no. of tags shown in bold (p < 0,05)
